# The Diagnosis, Treatment, and Clinical Sequelae of Sjogren’s Syndrome in a Pediatric Patient: A Case Report

**DOI:** 10.7759/cureus.38258

**Published:** 2023-04-28

**Authors:** Dakota C Davis, Meghan J Filson, Claire G Yother

**Affiliations:** 1 Medicine, Alabama College of Osteopathic Medicine, Dothan, USA; 2 Medicine, Edward Via College of Osteopathic Medicine, Auburn, USA; 3 Pediatrics, Gadsden Pediatric Clinic, P.A., Gadsden, USA

**Keywords:** autoimmune hepatitis in children, solid organ transplant, autoimmune syndromes, medicine-pediatrics, sjogrens syndrome

## Abstract

Sjogren's Syndrome is a chronic multisystem autoimmune condition where lymphocytes attack exocrine glands. Although this condition occurs in pediatric populations, it is often a missed diagnosis or diagnosis made after significant disease progression, frequently leading to extensive investment of time and resources. This case study follows a six-year-old African American female who, after an extensive medical course, was ultimately diagnosed with Sjogren's Syndrome. This case study intends to increase awareness of the potential abnormal presentations of this connective tissue disease in special populations, specifically school-aged pediatric patients. Even with the rarity of this condition in the pediatric population, physicians should keep Sjogren's Syndrome on their differential diagnosis when a patient presents with atypical or non-specific autoimmune-like symptoms. The presentation of children can be more severe than anticipated in an adult. A rapid, multi-disciplinary approach must be implemented to improve the prognosis of pediatric patients with Sjogren's Syndrome.

## Introduction

Sjogren's Syndrome (SS), known as dacryosialoadenopathia atrophicans, is a chronic multisystem autoimmune condition where lymphocytes attack exocrine glands [1]. SS commonly presents as a dyad of xerostomia and xerophthalmia secondary to lymphocytic invasion and subsequent destruction of the salivary and lacrimal glands [1,2]. Pathology is not limited to exocrine glands, as SS has also been found to impact other body systems, including pulmonary, renal, hepatic, and neurologic, to name a few [3]. It is typically diagnosed during the fifth and sixth decades of life in Caucasian women [1]. It is currently predicted to affect anywhere from 1:100 and 1:1000 adults making it the most common connective tissue disease [4]. Although SS is considered the most common connective tissue disease, the occurrence of this condition in pediatric populations remains a mystery. Although this condition has been reported in pediatric patients, the clinical course, morbidity, and mortality in younger populations are currently difficult to assess fully [5]. This difficulty is perceived to be secondary to atypical initial presentations, an absence of pathognomonic signs and symptoms, and an absence of diagnostic markers in many patients [3,4]. For example, a review of PubMed completed by the author revealed less than 200 available case reports of pediatric Sjogren's Syndrome, most of which were combined with another autoimmune process or presented with rare or unusual symptoms. Also, it can be difficult to confirm the diagnosis.

To confidently diagnose the condition, one must use a systematic and comprehensive approach that excludes possible alternative diagnoses. Case reports have shown a range of presentations from patients with a minimally symptomatic sequela while affecting others more severely [3]. Classic findings of this condition include all or some of the following: persistent dryness of the eyes or mouth, parotid gland enlargement, a dramatic increase in dental caries, or the presence of specific serologic assessments (i.e., anti-Ro/SSA, anti-La/SSB, rheumatoid factor (RF), antinuclear antibody (ANA), and hyperglobulinemia) [3]. Diagnosis solely based on these findings is ill-advised and objective confirmation of the pathology is required. The confirmation of a diagnosis of SS has two criteria: objective evidence of ocular or oral dryness or objective evidence of glandular parenchymal damage and serologic or histopathologic evidence of autoimmunity [3]. The gold standard in obtaining a diagnosis of SS is a biopsy of an affected gland. A successful biopsy of an affected gland which subsequently undergoes histopathological analysis revealing focal lymphocytic sialadenitis, is "the best diagnostic criterion for the salivary component of SS, in terms of its disease specificity, convenience, availability, and low risk" [6].

## Case presentation

A six-year-old African American female with a previous history of asthma presented to her primary care provider with a chief complaint of upper respiratory symptoms, including rhinorrhea, a minimally productive cough, and a fever. The patient was diagnosed with Influenza A and discharged home with a prescription for Oseltamivir (45mg twice daily PO). The patient's symptoms failed to improve, and her mother ultimately took her to the emergency department. She was diagnosed with an acute bacterial sinus infection and acute otitis media there. She was discharged home with an amoxicillin prescription (1600 mg/day PO), filled and initiated the following day. Following the emergency department visit, the mother reported that her daughter was acting differently from her normal self. The patient was described as "talking out of her head" with examples such as speaking about a girl in her home when only her mother and herself were present and misusing common household items such as mistaking a television remote for a telephone. She had also been removing all clothing to urinate. The mother also reported a new gait abnormality with intermittent falling. The patient did experience a fall and injured her lip, but there was no reported loss of consciousness or seizure-like activity. The mother continued to disclose that the patient was now favoring her right arm over her left, and she was advised to present to a higher acuity institution for further evaluation.

Upon arrival, the patient was tachycardic, tachypneic, and hypertensive. She was also found to be altered from baseline mental status, only knowing her name and date of birth. The patient was somnolent and difficult to rouse, only responding to sternal rub. Mother denies any significant history of easy bruising but does endorse daily nose bleeds for the past month, all resolved following sustained pressure. An emergent non-contrast head computed tomography (CT) scan was obtained, confirming the previously stated diagnosis of acute sinusitis without any further intracranial pathologies. A confirmatory Influenza A viral assay was obtained and found to be negative. A lumbar puncture (LP) was performed, and broad-spectrum prophylactic antibiotics (vancomycin (900mg/day IV) and piperacillin/tazobactam (4800mg:600mg/day IV) were initiated while awaiting results. LP was found to be within normal limits. A subsequent battery of laboratory evaluations was ordered, including complete blood count (CBC), coagulation studies, comprehensive metabolic panel (CMP), ammonia levels, and urinalysis (UA) with culture.

CBC revealed thrombocytopenia at 108x109/L and was otherwise within normal limits. Coagulation studies revealed an elevated prothrombin time (PT) (22.5 seconds), an elevated activated partial thromboplastin time (aPTT) (38.2 seconds), and an elevated international normalized ratio (INR) (2.0). CMP showed no acute electrolyte abnormalities with adequate renal function. Liver enzyme assays revealed transaminitis. Results included elevated total bilirubin (7.2mg/dL), alanine aminotransferase (ALT) (400 IU/L), aspartate aminotransferase (AST) (600 IU/L), and alkaline phosphatase (ALP) (520 IU/L). The patient's ammonia level was elevated to 137 µg/dL. Urinalysis was unremarkable, and culture was ultimately negative. Due to abnormal liver functioning, unremarkable serum salicylate and acetaminophen levels were ordered. The next steps included a CT of the abdomen and pelvis with and without contrast.

Additionally, a pediatric gastroenterologist was consulted and elected to proceed with Lactulose administration in an attempt to resolve hyperammonemia. The abdomen and pelvis CT revealed multiple liver lesions concerning cirrhotic processes. This confirmed the cause of altered mental status being hyperammonemia secondary to hepatic encephalopathy.

The patient was admitted to the hospital secondary to altered mental status, non-reassuring lab results, and the need for continued observation. Monitoring revealed unchanging laboratory studies with worsening mentation, and the patient was maintained on Lactulose. She was later transferred to the pediatric intensive care unit (PICU), where she was intubated for prophylactic airway protection. During PICU admission, the patient underwent three rounds of plasmapheresis to lower ammonia levels. Her fulminant liver failure failed to improve, and her neurologic deterioration worsened. The underlying cause of her acute liver failure with altered mental status remained elusive, prompting further assessment. ANA testing, immunoglobulin levels, and ultrasound-guided liver biopsy were evaluated. The results were a positive ANA, an elevated immunoglobulin gamma (IgG) level, and a liver biopsy consistent with autoimmune hepatitis. Following this diagnosis, she underwent a magnetic resonance imaging (MRI) study of the brain, which revealed diffuse cerebral edema and bihemispheric restricted diffusion within the white matter. No neuronal loss or anaerobic metabolism was reported. She then underwent long-term, continuous video electroencephalography (LT-EEG), which did not reveal seizure activity but demonstrated non-specific diffuse moderate to severe cortical dysfunction. The patient remained in fulminant liver failure with neurologic compromise, and she was ultimately enrolled for an emergent liver transplant. She successfully underwent duct-to-duct liver transplantation.

The operative course was complicated by prolonged intubation, postoperative retroperitoneal hematoma requiring exploratory laparotomy and subsequent evacuation, self-limiting acute kidney injury (creatinine peak of 1.9mg/dL), persistent hypertension, and persistent hyperglycemia. The patient did not return to her baseline neurologic status, and she was started on Tacrolimus for organ rejection prevention. While recovering from transplantation, the patient became febrile with complaints of joint swelling. Upon further investigation, the patient was found to be anemic, hyperglycemic, and hypertensive, prompting Rheumatology and Endocrinology consultations. Rheumatology completed an extensive patient analysis, and the primary SS diagnosis was made. Rheumatology concluded SS as being the trigger for the patient's autoimmune hepatitis. She was subsequently prescribed Rituximab and steroids for the treatment of this condition.

Shortly after the diagnosis of SS, the patient underwent an ultrasound-guided liver biopsy to assess for rejection. This biopsy revealed a recurrence of autoimmune hepatitis treated with steroids, and the patient would remain on steroids indefinitely. An additional ultrasound-guided liver biopsy was completed three years later, which revealed mild acute cellular rejection of the transplanted liver. This was treated with intravenous Solumedrol. The patient was described as "excessively tremulous and aggressive" during this time. This was believed to be an adverse reaction to Tacrolimus, and the patient was switched to Sirolimus. Using Sirolimus necessitates using adjuvant agents to prevent rejection, and she was started on Prednisolone and Mycophenolic acid for additional coverage. Endocrinology also assessed the patient and diagnosed her with steroid-induced diabetes mellitus and hypertension. Secondary to the necessity of steroids in this patient, pharmacological interventions, including Glucerna dietary supplements and amlodipine, were initiated to treat diabetes mellitus and hypertension, respectively. The patient would continue to experience additional complications from prolonged steroid use, including vitamin D deficiency and osteoporosis resulting in a stress fracture of the right tibia requiring orthopedic follow-up, steroid-induced hyperlipidemia treated with prescription-strength Omega three fatty acids, and cushingoid body habitus.

One year following the transplant, the patient started experiencing seizures. The diagnosis of localization-related symptomatic epilepsy syndrome with complex partial seizures was given. This is currently managed with Cannabidiol, Levetiracetam, Clobazam, Lacosamide, and Trazodone. One year after the transplant, the patient was readmitted with urosepsis secondary to Klebsiella pneumoniae. Approximately four years following her prolonged diagnostic workup and subsequent complications, the patient was rehospitalized for a second bout of urosepsis complicated by concurrent upper respiratory infection (URI). The patient's urinary tract infection (UTI) was treated appropriately with Ceftriaxone after urine culture results returned positive for Klebsiella pneumoniae again. She was prophylactically treated with Nitazoxanide for her URI resulting from norovirus. While in the hospital, the patient was found to be colonized with candida species and treated accordingly with 14 days of intravenous Micafungin. The patient did experience another acute kidney injury during this hospitalization with a creatinine peak of 2.2mg/dL. This was completely resolved before discharge from the hospital. Secondary to her infections and hospitalization, her immunosuppressive regimen consisting of Sirolimus, prednisolone, and mycophenolic acid was changed from 1mg to 0.75mg once daily, from 7.5mg to 3mg once daily, and 780mg twice daily to 500mg orally twice daily, respectively. These regimens were returned to their previous dosages before discharge home. The patient displayed no signs of acute or chronic cellular rejection of the transplanted liver during her hospital stay, as confirmed by an ultrasound-guided liver biopsy.

About two weeks after discharge, the patient was seen by her primary pediatrician for a 12-year-old well-child assessment appointment. Standardized comparisons of her growth were made against growth charts according to protocol. She continues to have global developmental delay, epileptic seizures, hypertension, diabetes mellitus, and hyperlipidemia. Physical exam was remarkable for a healthy and well-appearing wheel-chair-bound female with cushingoid habitus and five+ sustained beats of clonus to the bilateral lower extremities with three + reflexes throughout. She remained interactive regardless of neurologic deficits and displayed no obvious signs of depression, anxiety, altered mood, or hyperactivity. She is regularly followed by neurology, rheumatology, physical therapy, her transplant team, nephrology, endocrinology, and orthopedics, in addition to her primary care provider/pediatrician. The continued plan for this patient will remain reactionary as her needs arise. Due to the severity of her hepatic encephalopathy, she has been unable to and is not expected to achieve the appropriate mental and physical milestones.

## Discussion

Although SS is believed to be the most common connective tissue disease, this condition remains underreported in pediatric populations [5,7]. The Big Data Sjogren Project Consortium is an international multi-faceted organization encouraging data sharing from SS clinical databases across five continents [8]. A study was completed utilizing the 2002/2016 diagnostic criteria for SS and solely focused on individuals diagnosed with the condition before age 19 [8]. Of the 12,083 patients in the registry, only 158 (1.3%) had a childhood onset diagnosis, with a mean age of 14.2 years, with females affected more than males [8]. Of this 1.3% cohort, 80% reported xerostomia, 70% reported xerophthalmia, 33% had parotid enlargement, 97% had a positive minor salivary gland biopsy, and 94% had an abnormal salivary ultrasound study [8]. From a serologic standpoint, 90% were positive for ANA, 89% were positive for anti-Ro/SSA/anti-La/SSB antibodies, and 68% were positive for RF [8].

In summary, this study was revolutionary and truly showed the infrequency of SS in pediatric populations. Interestingly, pediatric patients with primary SS are frequently diagnosed in mid to late adolescence, while the subject of the above case study was only six years old. The good news is that when SS presents in mid to late adolescents, the clinical presentation is consistent with the presentation of an adult with SS; the same cannot be said for younger pediatric populations, further highlighting the need for the above case studies' representation in the medical literature. This case highlights the need for pediatric clinicians to have primary SS on the differential for pediatric patients presenting with non-specific autoimmune-like symptoms.

This case report highlights the uniqueness of an early pediatric presentation of SS and calls attention to better understanding this condition in school-aged pediatric populations. We believe the medical professionals involved in her case acted appropriately in achieving a diagnosis and formulating a comprehensive treatment plan based on the patient's presentation. The following will highlight and discuss pertinent points in her care to help medical professionals understand what actions to take if a similarly rare presentation occurs.

Her previously undiagnosed SS was ultimately concluded to have elicited autoimmune hepatitis. This pathophysiologic process was unfortunately undetectable until it was too late, resulting in an entirely reactionary plan of care for this patient. This process exacted a heavy toll and was incredibly costly physically, emotionally, and financially, further highlighting the need to understand better this connective tissue disease and its various presentations in unique populations. Her sudden onset and severity of presenting symptoms should be noted. The atypical clinical presentation and the inability of these patients to fit into the adult diagnostic criteria emphasize the difficulty of diagnosing a child with SS.

Secondary to the absence of diagnostic details in this patient's rheumatologic workup, a deeper dive into the diagnostic criteria of this condition is warranted. To reiterate, SS is diagnosed with either objective evidence of ocular and oral dryness or objective evidence of glandular parenchymal damage with serologic and histopathologic evidence of autoimmunity [3]. Objective ocular dryness can be confirmed with a Shirmer one test which has been utilized to assess the adequacy of tear production since its conception in 1903 [9]. This test is completed by folding a strip of sterile filter paper and placing it at the lower margin of each eye before prompting the patient to close their eye and measuring the extent of moisture obtained by the paper over 5 minutes [9]. Variations in this testing modality include the presence or abscess of anesthesia to the eye. The difference is that topical anesthesia in the eye allows for a better assessment of basal tear production, whereas an absence of anesthesia assesses reflex tearing of the tested eye [3]. Less than 5mm of moisture obtained in five minutes indicates tear production deficiency and is considered diagnostic for SS [10]. Recent advancements use ocular surface staining with Rose Benthal dye, fluorescein, or lissamine green to confirm ocular dryness. The results have been promising for the diagnosis of SS [11]. To quantify salivary hypofunctioning and subsequent oral dryness, one can use salivary gland scintigraphy or whole sialometry [3]. Salivary gland scintigraphy utilizes a radionuclide scan to assess major salivary gland functioning, revealing hypofunctioning glands as having low compound uptake [12]. Even though this test is only positive in about one-third of patients with SS, it is still considered specific yet insensitive, and some specialists prefer it over whole sialometry [13]. Whole sialometry remains another option for assessing oral dryness; however, multiple protocols have been published, the esoteric details of which lie outside this case study [14]. Evidence of glandular parenchymal damage can be ascertained through imaging modalities, including ultrasonography, magnetic resonance, and CT [3,15,16]. The next diagnostic criteria for discussion are serology and histopathologic evaluation. The pathognomonic serology for SS is the presence of either anti-Ro/SSA antibodies, anti-La/SSB antibodies, or a combination of both serologic markers, which are positively identified in approximately 60 to 80% of patients with SS [3]. These markers have a lower prevalence in the general population, supporting their use for diagnosing this condition [17,18]. Other diagnostic serological markers have been found while assessing patients with SS. The prevalences of these markers are not as highly associated with the disease but may provide an avenue for diagnosis in atypical cases and have even been associated with higher severity of this pathophysiologic process [19]. Finally, the most objective piece of diagnostic evidence for a patient to be diagnosed with SS is to perform a biopsy of an area with suspected involvement.

With a better understanding of the prevalence of this condition in pediatric populations and the diagnostic criteria of this condition, we can now discuss our patient's clinical course further. The patient's initial presentation mirrored that of a seasonal viral infection, which is expected in a pediatric patient. When adequate treatment was initiated, and the patient continued to decline, appropriate next steps were taken, and the patient's care was escalated to a higher acuity facility. Following this treatment chain and commenting on the potential differential diagnoses is important as her condition reveals itself. This pediatric patient presented to the emergency department with altered mental status (AMS). AMS encompasses many disease pathophysiologies. This is especially noted in pediatric populations, and the list of potential etiologies includes infections, metabolic derangements, trauma, toxic ingestions, and even psychiatric disturbances, to name a few [20]. The initial concerns for these etiologies are reflected in the patient's work which included interviewing the primary caregiver regarding changes in the patient's behavior, a head CT, an extensive laboratory workup with cultures as indicated, and a lumbar puncture. The lack of leukocytosis and fever made infectious etiologies less likely. The negative urine culture and unremarkable lumbar puncture further confirmed this. This patient was born in a hospital and had all necessary neonatal screenings completed and repeated at one month of age. If a metabolic derangement were suspected, this would have most likely been discovered via neonatal screenings or the patient becoming symptomatic much younger. A family history of negative for autoimmune conditions coupled with normal blood glucose levels and an absence of ketones in her urine made diabetic ketoacidosis less likely. Properly assessing parent-patient interactions and a negative head CT made non-accidental trauma less likely. Accidental ingestions were something that needed further elucidation. This was represented by ordering the patient's acetaminophen level within normal limits. The mother reports she had been paying close attention to the daughter the few days before the emergency department presentation and did not report any accidental ingestions. In the context of a recent suspected viral illness and subsequent altered mental status with elevated ammonia, a diagnosis of Reye Syndrome could not be excluded. Reye syndrome is an acute pediatric emergency consisting of hepatic dysfunction and noninflammatory encephalopathy, the hallmark of which is hyperammonemia and hypoglycemia [20]. This is hypothesized to be caused when viral syndromes are treated with salicylates in children, and the mainstay of treatment is entirely supportive, with lasting neurological deficits expected [20]. The patient's salicylate was 0, indicating she had no exposure to salicylate before her altered mental status and hyperammonemia.

 It was late in this patient's extensive diagnostic workup that autoimmunity panels were utilized, ultimately highlighting the etiology of her final diagnosis. Altogether, this patient presented with end-stage sequela of what started as non-specific autoimmune symptoms, thus prompting further investigation. It was not until the conclusion of this investigation that Sjogren's Syndrome was diagnosed, a condition which was not an obvious diagnosis and, therefore, not included in the initial differential for this patient. Due to the severity of this patient's presentation and subsequent postoperative complications, she now has additional diagnoses of epilepsy and global development delay due to hepatic encephalopathy, steroid-induced diabetes mellitus, hyperlipidemia, and osteoporosis due to her chronic requirement and use of immunosuppressive therapy, as well as hypertension and subsequent acute kidney injury secondary to hypoperfusion during her transplant procedure.

This case study intends to demonstrate an atypical and severe presentation of the most common connective tissue disease, Sjogren's Syndrome, in a unique patient population. The severity of this case can help bring awareness to the vast differences in the presentation of this condition in pediatric populations from the greater general population. In this case, we provide insight into how the sequelae of this condition may be avoided in the future.

## Conclusions

SS is believed to be the most common connective tissue disease in the general population based on the most recent epidemiologic data. Secondary to this disease's prevalence, many resources and diagnostic protocols have been developed to diagnose and further classify this condition in various patient populations. Due to previously discussed reasons, this diagnosis continues to remain elusive in pediatric populations. With the above documented aberrant presentation of this condition in a pediatric patient and literature supporting its complications with diagnosis in this population, physicians should keep SS on their differential diagnosis when a young patient presents with autoimmune-like symptoms without an easily identifiable cause. The presentation of pediatric patients with SS can be more severe than anticipated in adult populations, highlighting the need for rapid, multi-disciplinary consultation. Utilizing a multi-disciplinary team may be beneficial in improving diagnostic sensitivities and specificities and the potential prognosis for many patients to come.
